# Two-component systems regulate bacterial virulence in response to the host gastrointestinal environment and metabolic cues

**DOI:** 10.1080/21505594.2022.2127196

**Published:** 2022-09-24

**Authors:** Claire Shaw, Matthias Hess, Bart C. Weimer

**Affiliations:** a Department of Animal Science, Systems Microbiology & Natural Products Laboratory, University of California, Davis, USA; b Department of Population Health and Reproduction, 100K Pathogen Genome Project, University of California, Davis, CA, USA

**Keywords:** Metabolic cross talk, pathogenesis, host–microbe interaction, enteric bacteria, infection modulation

## Abstract

Two-component systems are ubiquitous signaling mechanisms in bacteria that enable intracellular changes from extracellular cues. These bacterial regulatory systems couple external stimuli to control genetic expression via an autophosphorylation cascade that transduces membrane signals to intracellular locations, thereby allowing bacteria to rapidly adapt to the changing environmental conditions. Well known to control basic cellular processes, it is evident that two-component systems also exercise control over virulence traits, such as motility, secretion systems, and stress responses that impact the complex cascade of networks that alter virulence traits. In the gastrointestinal system, cues for activation of virulence-related two-component systems include metal ions, host-derived metabolites, and gut conditions. The diversity and origin of these cues suggest that the host can exert control over enteric pathogenicity via regulation in the gastrointestinal system. With the rise in multi-drug resistant pathogens, the potential control of pathogenicity with host cues via two-component systems presents a potential alternative to antimicrobials. Though the signaling mechanism itself is well studied, to date there is no systematic review compiling the host-associated cues of two-component systems and virulence traits. This review highlights the direct link between the host gastrointestinal environment and pathogenicity by focusing on two-component systems that are associated with the genetic expression of virulence traits, and that are activated by host-derived cues. The direct link between the host gastrointestinal environment, metabolites, and pathogenicity established in this review both underscores the importance of host-derived cues on bacterial activity and presents an enticing therapeutic target in the fight against antimicrobial resistant pathogens.

## 1 Introduction

Bacteria respond and actively adapt to their surroundings through a variety of well-characterized sensing systems. These sensing mechanisms are essential for adjusting the cellular state in response to shifts in local conditions as these local changes require an immediate response for survival. One response mechanism that has been studied in great detail, and which we will focus on in this review, involves two-component regulatory systems (TCSs). TCS mechanisms are ubiquitous in bacteria and are key regulators involved in a myriad of cellular processes, such as growth, biofilm formation, virulence, and secretion [1–4]. In this system, external stimuli are detected via multi-domain membrane-based proteins that facilitate an intracellular response by transducing the external stimulus into an internal cascade of signals via their corresponding response regulator domain in the cytosol. Phosphoryl transfer between the two proteins subsequently triggers the activation or repression of the corresponding genes that change the cellular response. While the basic structure of TCSs is conserved, the ultimate effect of each system depends on the various molecular building blocks of the specific TCS (e.g. signal molecule, structure of sensor and regulator, and the genes that are activated or repressed). Whereas TCSs are involved in many different cellular responses, this review will focus primarily on TCSs that are involved in controlling virulence among enteric bacteria that reside in various locations within the digestive system. Though the characteristic response cascade of TCSs is well studied, to date there is no systematic review of host-derived activators of these signalling cascades in host-associated pathogenic bacteria.

This curated presentation of a focused subset of these signalling systems draws a direct link between host metabolic and gastrointestinal activity and the control of virulence in the gut, highlighting the possibility of TCSs as therapeutic targets for pathogenic control. This review concentrates particularly on TCSs related to bacterial virulence and that are regulated by conditions in the gastrointestinal tract, metabolic products, or key host-microbe nutrients (Table 1). Virulence-related TCSs for which the activation signal is not known or is not a host-derived cue will not be discussed here. Although the experimental verification of TCS activation by specific signals is understudied and therefore in some cases ill-defined, this review provides a valuable summary of some of the existing bacterial TCSs and highlights their prevalence and role in modulating the expression of genes that directly regulate virulence of enteric pathogens.

**Table 1.** Two-component receptor systems (TCS) sorted by signal type. TCS represented by the histidine kinase (HK) and response regulator (RR) pairs. Result of TCS may be due to either repression or activation post-signal response. Systems marked with * are highly conserved TCSs across genera.

	Signal	HK-RR Pair	Result	Genus
Metal Ions	Zn^2+^	ColS-ColR	LPS modification	*Pseudomonas* [5]
	Cu+/Ag+	CusS-CusR	Tripartite efflux pumps	*Escherichia [6], Klebsiella* [7]
	Mg^2+^	CsrR-CsrS	Activation of virulence repertoire	*Streptococcus* [8]
	Mg^2+^, Ca^2+^	PhoQ-PhoP*	LPS modification, Low Mg^2+^ adaptation, Antimicrobial resistance, PmrAB cross talk	*Escherichia [7], Salmonella [9], Yersinia [10], Stenotrophomonas* [11]
	Mg^2+^, Fe^3+^, Zn^2+^	PmrB-PmrA* BasS-BasR	Stress response, Antimicrobial resistance, PhoPQ cross talk	*Escherichia [7], Pseudomonas [12], Salmonella* [13]
Amino Acids	Glutamate	GluK-GluR	Antibiotic synthesis	*Streptomyces* [14]
	Glutamate, Aspartate	AauS-AauR	Swarming, Motility, Biofilm formation	*Pseudomonas* [15,16]
Amino Acid Derived Molecules	Serotonin (Via Tryptophan)	CpxA-CpxR	Type 3 Secretion System	*Citrobacter [17], Escherichia* [17]
	Epinephrine/Norepinephrine (Via Tyrosine)	QseC-QseB	Biofilm formation, Flagellar motility	*Escherichia [18], Haemophilus [19], Salmonella* [20]
	Epinephrine (Via Tyrosine)	QseE-QseF	Type 3 Secretion System	*Escherichia* [21]
	Nitrate/Nitrite (Via Arginine)	NarX-NarL NarQ-NarP	Motility, Biofilm formation, Nitrate Sensing and Reduction	*Burkholderia [22], Escherichia [23], Pseudomonas [24], Salmonella* [25]
Environment & Other	Acidic pH	EvgS-EvgA	Multidrug Transporter	*Escherichia* [26]
	Acidic pH	TrxR-TrxS	Biofilm formation	*Streptococcus* [27]
	Acidic pH	HK11-RR11	Biofilm formation	*Streptococcus* [28]
	Osmolarity	EnvZ-OmpR	Intracellular iron regulation	*Escherichia [29], Klebsiella [7], Pseudomonas [7], Yersinia [100]*

## 2 Host–Microbe interactions at the gastrointestinal interface

The digestive tract, and especially the colon, is an ecosystem with high microbial density and activity. For bacterial survival in these highly competitive ecosystems, a rapid, continuous and well-tuned response is needed to accurately adapt to changing, environmental, nutrient and metabolite concentrations. With the high microbial density and exposure to nutrients from the host diet, the gut microbiome contains not only commensals but also a diverse set of opportunistic pathogens, foodborne pathogens, and host adapted pathogens, making tight control of the microbiome composition via metabolism shifts a requirement to avoid dysfunction [30]. One mechanism for the regulation of microbial growth is achieved by sensing metabolites in the immediate surroundings and many of the metabolites that regulate the microbial response can also affect host health directly and indirectly. Indole, for example, is a bacterial product derived from tryptophan that is well known for its ability to control many aspects of bacterial physiology [31], and in the host indole promotes the formation of tight junctions in gut epithelial cells [32]. Though indole exerts a protective effect in the gut, other microbial metabolites derived from the same precursor, tryptophan, can affect host behaviour via interactions in the circulatory system and brain [33–35]. Since bacterial metabolites can affect host systems beyond the gut barrier, it is not surprising that metabolic products along with related systemic changes in the host can also affect the transcriptional regulation of bacterial genes, and therefore bacterial growth and pathogenicity directly in the gut. One integral link in these host activity-pathogenic activity interactions are the TCSs sensitive to host conditions and cues.

## 3 Two-component systems and virulence

**Figure 1.**
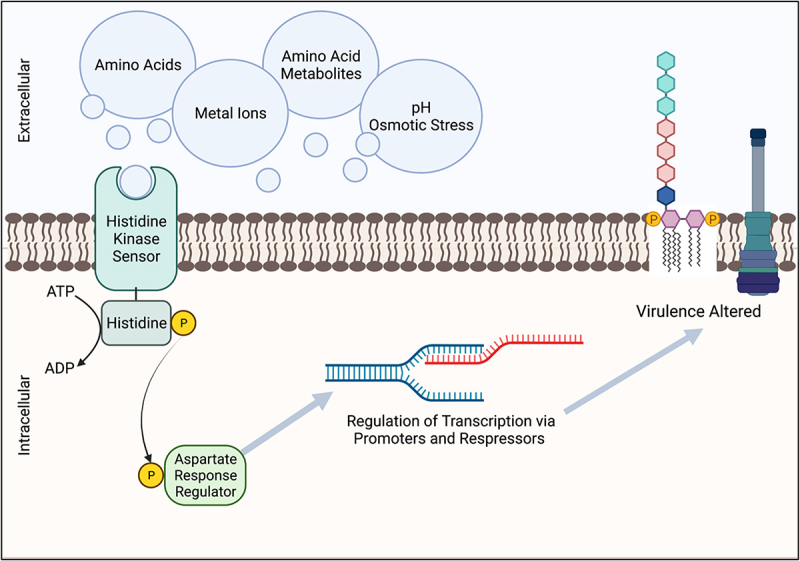
Two-component receptors systems (TCS) related to virulence can be modulated by host-derived metabolic products. Signaling molecules are sensed via the multidomain surface membrane protein histidine kinase (HK), which is then undergoes autophosphorylation. The phosphoryl group is then transferred to an intracellular aspartate residue response regulator (RR). Transcription of the corresponding genes is then repressed or activated, dependent upon the originating TCS and signal. Virulence factors controlled in part by TCSs include LPS modifications for decreased host detection and upregulation of type 3 secretion systems (T3SS).

Two-component systems share a common overarching transduction method to translate extracellular signals into intracellular actions via phosphorylation. During the initial step of this cascade, a membrane-bound histidine kinase (HK) senses changes in extracellular conditions (e.g. pH, osmolarity, metal ions, nutrients, metabolites) and autophosphorylates at the histidine residue using an ATP molecule (Figure 1). This change at the HK is relayed to a cognate response regulator (RR) via subsequent phosphorylation of an aspartate residue of the RR (Figure 1). The activated intracellular RR then elicits the corresponding cellular action, typically, the induction or repression of a gene or sets of genes that are involved in complex pathways (Figure 1). Response regulators in the TCS cascade can change the cellular response via direct transcriptional regulation, but the role of RRs is diverse and in some cases they also exercise control over the activation of other TCSs or are involved in other protein–protein interactions [36]. Two-component systems across prokaryotes share core signalling properties and often high sequence similarity [36], but small changes in the receiver or effector domains of these signalling proteins results in distinct activity and response, giving rise to the observed TCS variety amongst prokaryotes. Given their ubiquity and integral role in controlling bacterial activity, the commonalities of TCSs have been extensively reviewed and more details regarding the mechanisms of these systems can be found in other reviews [4,37,38].

Signalling systems, like the TCS that display a multi-step relay that amplifies extracellular signals have been observed across different phylogenetic groups of bacteria. They were observed for the first time in *Escherichia coli* four decades ago [39] and there is now strong evidence of TCS-related proteins in all kingdoms of life, except in animals [40]. TCSs regulate many bacterial activities, ranging from basic growth control to chemotaxis to pathogenic capability. Given the wide range of processes that TCSs control, it is unsurprising that single cells can possess multiple different histidine kinase/receptor protein sets. *E. coli*, for example, is known to have at least 29 TCS sets (HK/RR pairs), while *Salmonella enterica* serovar *Typhi* has 30 HK/RR pairs, and *Pseudomonas aeruginosa* has at least 64 genes that encode distinct response regulators and 63 genes for different HKs, the most TCS pairs that have been reported in one bacteria to date [41–43]. The prevalence of these signalling systems coupled with continuously increasing whole bacterial genomes has led to the development of methods for predicting the target genes of TCSs in prokaryotes [44]. Predictive methods, coupled with experimental confirmation, have expanded our overall understanding of TCSs and continually introduce potentially novel mechanisms for the control bacterial activity. The ubiquity and continued expansion of known TCSs together indicate that these systems may offer new targets to control bacterial infections and ultimately, perhaps, become an antibiotic alternative to control infection [45].

TCSs facilitate the tight control of basic cellular processes, and while many of the known TCSs can be critical to cellular survival, none may be more relevant to medical applications than those that regulate virulence [46]. As with many cellular processes, individual TCSs are not the sole determinant for virulence. Instead, a combination of environmental triggers, differing HK sensor domains, and a vast network of response regulators often work simultaneously to promote or repress virulent traits. The bacterially dense and diverse human gut microbiome is rich in known and yet to be discovered TCSs [7]. Pathogens, whether opportunistic full-time residents of the human gut or transient pathogens, rely on exogenous stimuli that indicate ideal conditions for deployment of virulence traits (e.g. flagellar movement, biofilm formation, secretion systems, and metal sequestration) during the onset of disease initiation [47–49]. Coordinated expression of virulence genes is essential for the adaptive advantage of the pathogen, since upregulation of these virulence genes in suboptimal conditions leads to inefficient use of cellular machinery, while downregulation of these genes when conditions are optimal would equally represent an evolutionary disadvantage for the pathogen’s persistence [50,51]. The balanced regulation of virulence genes requires sensitive and strong signalling cascades, which is something that TCSs, especially those that are sensitive to multiple cues, are capable of doing.

Biomolecules (e.g. amino acids) and cofactors (e.g. metal ions) play a role in virulence regulation via interaction with TCSs and metabolite-TCS interactions have been described for a variety of organisms in the gut, including *Pseudomonas aeruginosa* and *Escherichia coli* [41,52]. The interplay of host and microbe in the context of microbial pathogenesis and metabolite-TCS interactions presents an interesting but vastly understudied area that brings about an opportunity to modulate virulence using non-antibiotic approaches in the complex gut microbiome. Recognition that small molecules are primary drivers of the complex host-microbe interplay further supports the concept that the actions of both the microbiome structure and the host metabolism are important in promoting and maintaining microbiome diversity and ultimately host health.

## 4 Host metabolic products and conditions as TCS triggers

### 4.1 Metal ions

Metal ions are imperative to basic functions for host and microbes but can also be toxic once they exceed critical concentrations, making metal consumption a delicate balancing act. Metal requirements bring a particularly daunting challenge when balancing dietary metal acquisition to sustain basic processes like iron-dependent haemoglobin production and to support the gut microbes’ metabolism, while avoiding accumulating metals at toxic levels or leaving extra metal ions circulating for pathogen utilization via siderophores [53]. Presented with pathogenic challenges, the human immune system will undergo nutritional immunity, a metal sequestering event prompted by inflammation [54]. In opposition to host protections, and as co-evolving interactors with the host, microbes have evolved their own mechanisms for metal sequestration, co-opting, and utilization. The first step in the activation of these systems is the activation of a metal-responsive HK sensor. Metal responsive TCSs (e.g. CoIRS, CusRS, PhoPQ, CsrSR) are extremely diverse among organisms and can lead to varying phenotypic changes and impact on virulence. A brief description of how metals interact with different TCSs is described below.

Iron is well known as both a participant in diverse cellular processes and as a regulator of immunity and virulence. The BasRS system in *E. coli* is iron sensitive, promoting lipopolysaccharide (LPS) modifications via activation of the stress response and inducing polymyxin resistance [55]. BasRS is also involved in the bacterial response to metal toxicity, activating the production of membrane metal transporters [55]. This same TCS in *E. coli* responds to mild acidic conditions by inducing the *aceF* and *aceE* genes, which are both part of the pyruvate-dehydrogenase operon, ultimately resulting in increased acetate metabolism [55]. It should be noted that the BasRS TCS in *E. coli* is homologous to the PmrAB system, later discussed in the context of *Salmonella enterica* serovar Typhimurium and LPS remodelling.

Zinc is another metal, like iron, known to be important in the regulation of the immune system and pathogenic activity. The ColRS two component system, found in *Pseudomonas aeruginosa* and *Pseudomonas putida*, responds to the presence of Zn^2+^ and induces changes in LPS [5,56]. *P. aeruginosa*, a nosocomial pathogen, is an adaptive and hardy drug-resistant pathogen that relies on its LPS to survive and evade host defences [57,58]. ColRS contributes to this LPS-related adaptability, as activation of this TCS by Zn^2+^ promotes changes to the lipid A structure, the common target of antibiotics, contributing to *P. aeruginosa* survival and evasion of treatments [5]. EptA_pa_ is pEtN transferase that can modify the 4’ phosphate group of lipid A in *P. aeruginosa* and is under transcriptional control, at least in part, by the ColRS TCS [5]. In the presence of exogenous Zn^2+^, the ColS HK activates the ColR response regulator which then upregulates *eptA*_*pa*_ and leads to the remodelled and more resistant lipid A structure [5]. In conjunction with the upregulation of the pEtN transferase protein, the production of a different modifying protein, aminoarabinose transferase (ArnT), is decreased as a result of the ColRS cascade [5]. The upregulation of one transferase and simultaneous downregulation of another by the same TCS signal indicates both that the *P. aeruginosa* maintains tight control of LPS modifications in response to environmental conditions and that the ColRS system exerts multifaceted control over what appear to be singular endpoints, like the remodelling of LPS.

Just as zinc is sequestered by the host and co-opted as a cue by microbes, copper (Cu) is also part of the immune system’s antimicrobial arsenal, shuttled to the sites of invasion to cause bactericidal metal toxicity [54]. As with other metals, some bacteria have evolved mechanisms to overcome the host’s immune system response to sequester metals. In the case of Cu, *E. coli* and *Klebsiella pneumoniae* evolved the CusRS system [6,7]. The CusS HK senses Cu+ and Ag+ and relays that to the internal CusR response regulator, which in turn activates transcription of the *cusCFBA* gene cluster. This operon encodes for a tripartite efflux pump that allows for the removal of metal ions when the concentration exceeds the cellular threshold. This TCS allows *E. coli* and *K. pneumoniae* to dodge the host’s attempt at bactericidal control and thus allows for further growth and invasion leading to a more favourable environment for the pathogen to cause disease.

Magnesium, much like zinc and copper, is essential to basic functions in both host and microbe. Mg^2+^ serves as a cofactor for over 600 enzymatic reactions, is important in cellular proliferation, and is involved in the excitation-relaxation coupling cycle [59]. Although Mg^2+^ is critical to many basic processes, this metal ion is not well absorbed in the gut with its uptake primarily controlled by the kidney. The lack of active Mg^2+^transport from the gut lumen makes this mineral more available for bacterial use and it is therefore not surprising that numerous microbial Mg^2+^ transporters and sensing systems exist [60]. *Streptococcus pyogenes* encodes its own Mg^2+^ regulated TCS, CsrSR, which interfaces with up to 15% of the *S. pyogenes* genome, including genes related to phagocyte resistance and increased invasion [8]. CsrSR shares some structural similarity with the TCS PhoPQ, discussed below [8] and it is possible that CsrSR functions similarly to PhoPQ or even resulted from a common evolutionary ancestor. CsrSR co-opts the host immune response to optimize pathogenesis, enhancing resistance to phagocytosis, the primary host defence for group A streptococci via sensing host antimicrobial peptides, such as LL-37 [8]. In contrast, Mg^2+^ appears to decrease this resistant phenotype, perhaps conserving cellular energy for more ideal conditions [8]. In response to low Mg^2+^ concentrations, the CsrSR system in *Streptococcus* is inactive and the capsule genes regulated by CsrSR, *hasABC*, remain available for transcription [61]. The inactivation of CsrSR in response to low Mg^2+^ concentrations is in line with host physiological conditions, as the low ionic concentrations that repress the CsrSR TCS are typical of host extracellular circulating fluids, and so production of the protective hyaluronic capsule, repressed by activation of CsrSR, remains important to pathogenic success [61]. Conversely, higher Mg^2+^ activates the CsrSR cascade and in turn represses the capsule genes (*hasABC*) and other pathogenic secretion product genes (*ska* and *sagA*) [61]. The sensing of Mg^2+^ concentrations by CsrSR in *P. aeruginosa* confers to the pathogen the ability to determine whether the cell is within a host (low Mg^2+^ concentrations) or in a less-favourable environment for pathogenesis like soil or waste water (higher Mg^2+^ concentrations). This ability to sense environment confers a competitive advantage through the ability to deploy energetically taxing virulence mechanisms primarily in locations of likely success. The CsrSR TCS is less well characterized than other highly conserved TCS but does appear to contribute to virulence in group A streptococci via the sensing of Mg^2+^ and a host produced antimicrobial peptide.

Mg^2+^ is also tightly connected with virulence in multiple gut-based pathogens, including *Salmonella enterica* serovar Typhimurium [60]. Two systems that use Mg^2+^ as a signal related to virulence, PhoPQ and PmrAB, are highly conserved and appear in many bacterial genera, further highlighting the integral and evolutionary role of magnesium in cellular function [7,9–13,62]. Besides Mg2+, Ca^2+^, Fe^3+^, low pH and antibiotics can also activate these systems. When experimentally inactivated, the PhoPQ system in *Stenotrophomonas maltophilia* is unable to respond to the typical low Mg^2+^ environmental cue, resulting in increased susceptibility to β-lactam antibiotics [11]. *Salmonella* species with experimentally inactivated PhoPQ system possess decreased virulence, indicating that the PhoPQ TCS is intergral to virulent *Salmonella* phenotypes [9]. Another conserved TCS that is often found in conjunction with PhoPQ, the PmrAB system can cooperatively interact with the PhoPQ TCS. In *S*. Typhimurium, PmrAB activation upregulates the *pmrHFIJKLM* operon, leading to LPS remodelling via 4-aminoarabanose addition [13]. LPS restructuring reduces the binding ability and thus effectiveness of certain positively charged antimicrobial peptides, though the activation of *pmrAB* related genes may also make *S. enterica* serovar Typhimurium more susceptible to other antimicrobial peptides [13]. Though universal protection against antimicrobials is not conferred via a single TCS cascade, TCS-induced LPS alterations can reduce pathogen susceptibility to certain antibiotics, as is the case with the PmrAB TCS in *P. aeruginosa* and the subsequent decreased vulnerability to colistin When PmrAB and PhoPQ are both present in an organism they interact by transcriptional regulation; with PhoPQ activating the PmrAB TCS via the PhoPQ-regulated *pmrD* gene [13]. *S. enterica* serovar Typhimurium has both these systems and though PmrAB is directly dependent on PhoPQ for activation, the result of these two TCSs are in conflict [13]. More specifically, *S. enterica* serovar Typhimurium PhoPQ knockouts showed decreased resistance to antimicrobials while PmrAB knockouts displayed increased antimicrobial resistance [13]. The resistance phenotype in the case of these two TCSs is due in part to alterations in the rigidity and permeability of the cell membrane, with PhoPQ resulting in a more rigid, less permeable exterior and therefore a more resistant phenotype [13]. The contrasting cellular outcomes between PhoPQ and PmrAB, especially considering the known dependency of PmrAB on PhoPQ activation, highlights the importance of understanding the cellular response to not just singular TCSs but also the results of multiple or interacting TCSs within a single organism. For a bacterium being able to rapidly respond to continuously changing environments, having multiple TCSs work in tandem results in a more fine-tuned response to the presence/absence of external cues like antibiotics, which in turn allows for a quick and customized adaptation to a wide range of conditions via multiple convergent expression pathways.

### 4.2 Amino acids

Amino acids are key metabolites for host and microbe health, as both require amino acids to support protein catabolism, basic metabolic pathways, and fundamental cellular processes. Just as humans lack the ability to synthesize essential amino acids, some bacterial genomes lack genes required to catabolize certain amino acids [63]. While this tug-of-war over amino acids also makes them crucial to some host defence mechanisms, where the host attempts to sequester amino acids, bacteria have equally evolved mechanisms for efficient amino acid uptake and utilization [63]. TCSs, like GluKR and AauRS, play a role in the amino acid battle as sensors that use amino acids to regulate the genes and thus phenotype of bacteria [14,64].

In addition to the role of glutamate in central metabolism, glutamate is well known for its essential role in the glutamate-dependent acid resistance pathway, a bacterial defence mechanism against host acidification [63]. *Streptomyces coelicolor* is a soil-dwelling bacterium and is not known as a prolific human pathogen, but since *S. coelicolor* is a member of the *Streptomyces* genus, it is possible that its TCS cascades may also be found in other *Streptomyces* species that are known human pathogens. The genome of *S. coelicolor* encodes GluRK, a TCS that responds to glutamate [64]. In *S. coelicolor* it appears that circulating glutamate also regulates the glutamate uptake system and synthesis of some antibiotics [64]. Upon phosphorylation via the GluK sensor, GluR activates the glutamate uptake cluster *gluABCD* via direct binding of the response regulator to the promoter region [64]. In addition to the increased production of glutamate transport proteins, previous work on glutamate-related TCSs indicate that genes involved in glutamate metabolism may also be directly under the control of this TCS, though that was not confirmed in this study [64]. More directly related to virulence traits, the GluRK TCS promoted the expression of genes encoding several antibiotics like actinorhodin, undecylprodigionsin, and type-1 polyketide [64]. The increased expression of antibiotics upon GluRK activation with glutamate appears to be an independent cascade from that to produce glutamate transporters [64]. The antibiotic regulation is controlled through secondary signals and not through direct binding between the promoter region and GluR [64]. Glutamate is central to bacterial metabolism and exerts control over the virulence-related stress response in many bacteria [63]. The control of antibiotic expression also appears to be peripherally controlled by glutamate in the host-controlled environment, through signalling mechanisms adjacent to the GluRK TCS [64]. Though GluRK is currently only proposed in the conext of *S. coelicolor*, the central role of glutamate in enteric pathogens suggests GluRK or a similar TCS may also be found in human pathogens as well. To date, studies on the GluRK system are limited and additional work in this area might provide promising new insights into the biological role of this system in enteric pathogens.

As with *S. coelicolor* and the GluRK system, the AauRS complex in the plant pathogen *Pseudomonas syringae* and opportunistic human pathogen *P. putida* may provide an opportunity to shed light on TCSs in *Pseudomonas* species that are human pathogens, as many of these receptor systems appear to be conserved across multiple genera [7,15,16].

**Figure 2.**
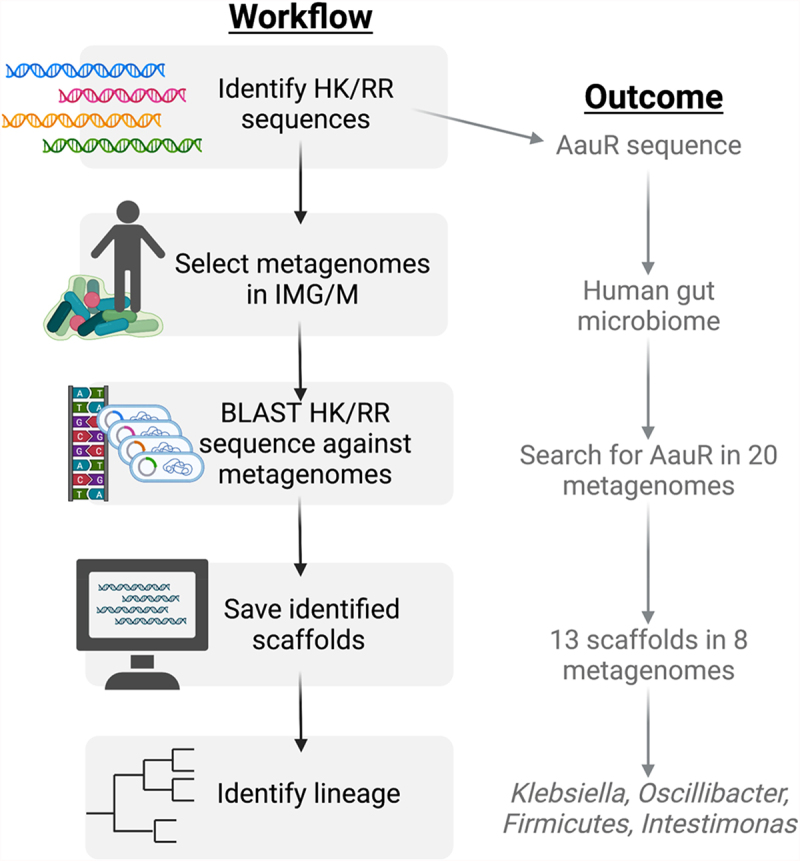
How to search for two-component systems in ecosystems via metagenome-specific BLAST. Known histidine kinase and response regulator sequences can be found via multiple genetic databases, including NCBI GenBank. Publicly available host and ecosystem-specific assembled metagenomes can be found on the Joint Genome Institute’s Integrated Microbial Genomes and Microbiomes (IMG/M) platform. BLAST searches with imported HK/RR sequences restricted to specific metagenomes can be done via the IMG/M platform. Search hits in the form of metagenome scaffolds can be saved and analysed for lineage and further analysis. This platform was used to search for the AauR response regulator in the human microbiome. 20 different human microbiome metagenomes were used as the search parameters, out of which 8 returned hits for the AauR regulator. The results were further narrowed into 4 different taxa: Klebsiella, Oscillibacter, Firmicutes, and Intestinimonas.

The AauS histidine kinase senses extracellular aspartate and glutamate, then activates the *aatJ* region via the response regulator AauR, all together upregulating amino acid uptake systems alongside activation of Type 3 Secretion Systems (T3SS). Together this cooperatively increases the virulent phenotype and therefore the risk to host [14]. Interestingly, the relationship between the AauRS system and the T3SS appears to have an evolutionary history, as this combination co-evolves and is conserved across at least 17 organisms [65]. The AauR response regulator, discussed here in the context of the plant pathogen *P. syringae*, can also be found in the metagenome of human microbiomes (Figure 2). The AauR regulator was identified in the genome of members from *Klebsiella, Oscillibacter, Firmicutes* and *Intestinimonas*, indicating that the gut microbiome likely harbours these vital signalling systems in a multitude of organisms for which they are not currently experimentally confirmed. Continued community contribution and careful curation of genetic databases, such as the ones highlighted in Figure 2, is vital for the expansion of TCS knowledge and for the implementation of these systems as therapeutic targets, especially as provided by analysis of the ever-expanding microbiomes that are available.

### 4.3 Amino acid metabolites

Just as amino acids play key roles in host–microbe interactions, so do their subsequent metabolic products. As central metabolism substrates, amino acids are responsible, or at least have connections, to almost all major metabolites. Aromatic amino acid metabolites include neurotransmitters (NTs), such as epinephrine, a tyrosine derivative, and serotonin, a tryptophan derivate, which are of particular interest since they have multiple important activities in the host [66,67]. Not limited to host production or use, neurotransmitters can be produced, utilized, and co-opted by the microbiome, sometimes to the benefit and sometimes to the detriment of the host [68]. In the context of TCSs, neurotransmitters are sensed by bacterial adrenergic receptors, which then relay the external stimulus to the response regulator and corresponding genes, regulating the virulent phenotypes of pathogens, just as done with metals. QseBC and QseFE both use epinephrine as an interkingdom signal for sensing the host condition and subsequently to increase virulence potential within the host gut [20,21,69,70]. The QseC sensor is a bacterial adrenergic receptor found across a diverse set of genera and is often linked to virulence, suggesting that the QseC sensor has been conserved over many generations or perhaps co-evolved with the host neurotransmitters [20]. Beyond amino acid-derived neurotransmitters, the QseC sensor is responsive to endocannabinoid metabolite, 2-arachidonoylglycerol (2-AG) [18]. The endocannabinoid system is beyond the scope of this review but recent work has linked the QseC sensor and LEE pathogenicity island, both responsive to amino acid-derived metabolites, to host-derived endocannabinoid molecules and so will be mentioned briefly in this section. Also discussed in this section is another amino acid-derived neurotransmitter, nitric oxide (NO), and its related metabolites, nitrite (NO_2_-) and nitrate (NO_3_-), which exert control over motility and biofilm formation in multiple pathogens [71]. Central to many metabolic routes in both the host and microbe, amino acids serve as precursors to many bioactive molecules. A few of these amino acid derived bioactive molecules are covered here in their relation to the control of virulence through interactions with TCSs.

In most cases, the response to TCS activation by host NTs results in the upregulation of secretion systems, increased motility, drug resistance and biofilm formation [12,19–21,69,70,72]. The utilization of host NTs as cues to increase pathogenic phenotypes is not surprising, as the NTs signal a state of stress and thus to bacteria signal a weakened host and ideal conditions for pathogenesis. As neurotransmitters of the sympathetic system, the nervous system involved in the stress response of the host, epinephrine and norepinephrine levels are moderately elevated in response to chronic stress and significantly elevated in response to acute stressors [73]. Disease states, whether chronic or acute, are arguably activators of the physiological stress response, a concept supported by the observable increase of circulating epinephrine in patients with Crohn’s Disease and Irritable Bowel Syndrome [74]. The QseC sensor uses elevated epinephrine levels to trigger pathogenesis in the gut. The utilization of this stress-related host NT is likely of evolutionary advantage to the pathogen as increased epinephrine levels may correspond in some cases to weakened host defences.

Serotonin is produced by the host, both by raphe nuclei in the brainstem and by the gut epithelium [75,76]. Additionally, the microbiome can produce this from the essential aromatic amino acid precursor tryptophan as well as respond to this compound as a ligand. Opposite to the role of epinephrine and norepinephrine, serotonin appears to decrease the pathogenicity potential of enterohemorrhagic *E. coli* (EHEC), as illustrated in mice using the model organism *Citrobacter rodentium* [77]. *C. rodentium* and *E. coli* rely on the locus of enterocyte effacement (LEE) pathogenicity island for the primary virulent apparatus, the T3SS, and the concomitant effectors that are injected into the host cell [77]. Increased luminal serotonin levels cues the CpxA HK to decrease the phosphorylation of the corresponding response regulator CpxR, *which results in a decreased level of transcription of the LEE pathogenicity island* [77]. The repression of LEE in turn decreases the virulent phenotype of *C. rodentium* and lowers the pathogenic load in the gastrointestinal system [77]. Conversely, lower concentrations of luminal serotonin levels do not actively repress the CpxR regulator, allowing active transcription of the LEE island and expression of the related virulence factors [77]. The CpxRA TCS is also sensitive to indole, another tryptophan-derived metabolite and one that is solely produced by bacteria. Indole in the large intestine forms a gradient with high luminal indole levels that decrease towards the epithelial layer [17]. Higher indole levels, like those seen in the lumen, repress the CpxRA signalling cascade, thereby decreasing the expression of virulent traits encoded by the LEE pathogenicity island [17]. In contrast, the low indole concentration observed at the epithelium activates the CpxA HK in the membrane of *C. rodentium* and leads to the phosphorylation of response regulator CpxR [17]. CpxR then promotes the transcription of the T3SS and cofactors, ultimately increasing the virulence of *C. rodentium* when in close proximity to the epithelium [17]. The sensitivity of CpxRA to both indole and serotonin provides flexibility for enteric pathogens encoding this TCS and allows for more tailored responses that are not only targeted towards the host gastrointestinal system, but to specific regions. Serotonin and indole are both tryptophan (Trp) catabolites and both exert some control over the virulent phenotype of pathogens in the gut, indicating that other Trp derived compounds may also display similar bioactive capabilities. Further work to examine the details of Trp catabolite actions between the host and the microbiome is needed for examination of the complex interplay between molecules that both host and microbe produce but utilize very differently.

The QseC sensor and the LEE pathway, both mentioned above in relation to different amino acid derivatives, converge in the context of the host-derived endocannabinoid metabolite, 2-arachidonoylglycerol. Though not an amino acid derivative itself, 2-AG is a host-derived metabolite that is an antagonist for the QseC receptor in EHEC and *C. rodentium* and thus is important to mention in order to highlight the diversity of cues and responses, even within the context of a single sensor [18]. 2-AG attenuates the virulence of EHEC and *C. rodentium* via modulation of the LEE pathogenicity island, which is described in more detail above [18]. The endocannabinoid system is a known neuromodulatory system that plays a crucial role in controlling the most basic physiological and homoeostatic processes [78]. The interaction between endocannabinoid metabolites and the virulence-modulating sensing systems of enteric pathogens is an emerging field and one that should be explored further, especially in light of the central role endocannabinoids play in systemic host physiology and disease [78].

Arginine, like the aforementioned amino acids tyrosine and tryptophan, is a key precursor to bioactive metabolites that serve as cues for virulence-related TCSs in enteric pathogens. Nitric oxide is a neuroactive molecule derived from arginine that is responsible for the regulation of host physiology, as with its role in peripheral vasodilation, and has emerging roles in the modulation of neurological disorders like Schizophrenia [79,80]. Bioactive NO can be produced from exogenous and endogenous arginine via nitric oxide synthase (NOS) activity, or from nitrite (NO_2_-) and nitrate (NO_3_-) [81]. Nitrite and nitrate, together with nitric oxide make up the nitrate-nitrite-NO pathway, a dynamic pathway involved in regulation of the cardiovascular system, the modulation of metabolic functions, and the control of inflammation [82]. Nitrite and nitrate are bioactive molecules, able to regulate microbial metabolic and pathogenic activities via the NarLX and NarPQ TCSs, and can be derived from leafy greens in the diet or from the breakdown of arginine [71,82].

As a review by Rocha et al. highlights, the broad effects of nitrate and nitrite on the gut microbiome at large remain somewhat obscured by the complexity of tracking the source and the route of these multitasking substrates throughout the host [83]. Some research indicates that nitrate does alter the composition and activity of the gut microbiota in disease states, modulating the nitrate-nitrite-NO pathway, improving dysbiotic conditions [84]. Though the mechanisms of generalized control of gut microbiome activity and diversity by nitrates remains somewhat unclear, the direct interaction of nitrate and nitrite with specific gut pathogens is well understood to be regulated by the TCSs NarLX and NarPQ [85]. NarLX and NarPQ, both involved in nitrate/nitrite sensing, communicate via crosstalk between sensor NarQ and response regulator NarL [24,86]. In *P. aeruginosa*, NarLX knockouts display increased biofilm formation in conjunction with decreased motility [85]. Mutants with only response regulator NarL attenuated but functioning NarX sensors displayed opposing activities, with decreased biofilm formation and increased swarming [85]. Both NarLX effects can likely be attributed to the control of rhamnolipid production, a key glycolipid that acts as a biosurfactant and is an antibiofilm compound [85]. Nitrate metabolism in *P. aeruginosa* is central to the pathogen’s success in the gut, as nitrate can be used as a terminal electron receptor for respiration and the regulatory glycolipid, rhamnolipid, is derived from nitrogen metabolism [85]. *E. coli* uses nitrate and nitrite in a similar way to *P. aeruginosa* and relies on the NarLX TCS to indicate environmental nitrogen conditions to trigger targeted cell responses [23,24,87]. *E. coli* additionally uses the NarPQ TCS to sense nitrate in the local environment [24]. The two TCSs interact with NarQ activating regulators NarP and NarX, and NarL activation serves as an inhibiting signal for NarQ-NarX crosstalk [24]. A recent study connected NarL activation by nitrate to mitigatation of the production of CgsD, a protein that contributes to the development of biofilms in *E. coli* [88]. The link between NarL and biofilm formation is apparent in multiple pathogens and suggests a clear bacterial signalling and activity cascade: the activation of NarL by nitrate is a positive indication to the cell of sufficient exogenous nitrogen sources for pathogenic growth, and therefore the protection biofilm formation provides is not necessary for survival in these nitrogen sufficient environments. Similar biofilm control by the NarLX/NarPQ TCSs can be seen in human pathogen *Burkholderia pseudomallei* [71,89]. As nitrate and nitrite control and metabolism is central to many cellular processes, it is not surprising that many symbiotic and pathogenic bacteria have one or both of the previously described nitrogen-sensing TCSs or a homolog of these TCSs [22,25,90], though more detailed mechanistic research is necessary to link these nitrogen related TCSs directly to the modulation of virulence in more pathogens.

### 4.4 Environmental changes and other signals

The gastrointestinal environment is a tightly regulated ecosystem with a network of epithelial transporters, sensors, and feedback loops for the management of nutrient reabsorption, acidity, and immune responses. The gastrointestinal environment and its associated microbiome, while under tight control, displays temporal (via diet and host metabolism), longitudinal (across compartments), and spatial (from lumen to mucosa) succession. As with specific metabolic products, some histidine kinase receptors can sense broader signals, such as fluctuating pH and osmolality. EvgAS in *E. coli* responds to an acidified environment, with moderate regulation at pH   5.5 and increased response at pH2.5 [91]. Activation of the EvgS HK in acidic conditions upregulates the *E. coli* survival-activating promoter AR2 via the EvgA response regulator, leading to increased acid resistance via the stringent response [91]. As with many TCS systems, it remains unclear whether EvgAS senses the pH drop exclusively, or whether there is a combination of factors that activate this sensor in cooperation with pH changes and other stress conditions. EvgAS is a conserved system across *E. coli* genomes and its behaviour varies in response to stress signals, with some evidence that EvgAS does not act alone to activate the stress response to alter the TCS activation [26]. For example, the well-conserved metal-sensing *phoPQ* operon encodes for a stress sensing TCS, and PhoPQ appears to act in tandem with EvgAS, perhaps even through direct signalling between these two TCSs [26]. Further complicating the regulatory cooperation, EvgAS is inhibited by indole [91]. In the presence of indole, the AR2 promoter is downregulated, subsequently silencing the stress response in *E. coli* [91]. The opposing effect of indole on the EvgAS system is partially explained by indole’s role as an intracellular pH regulator and as indole’s alternate activity as a bacterial signalling molecule that changes many phenotypic traits important to persistence and infection. Downregulation of the metabolically and energetically taxing cellular stress system in the presence of increased bacterial neighbours may be optimizing *E. coli*’s growth patterns in complex environmental conditions. The multiple activities of the Trp-derived catabolites as ligands and multi-functional modulators complicates the regulatory dynamics of the TCS and the highly flexible cellular response.

TrxSR is one of 13 TCSs identified in *Streptococcus* and one of three TCSs known to regulate virulent phenotypes. Much like EvgAS in *E. coli*, TrxSR responds to an acidified extracellular environment, and promotes the multiple gene activator (*mga*) virulence regulon, ultimately promoting quorum sensing and biofilm formation [92]. One of the other three virulence TCSs in *Streptococcus* is HK/RR11, an additional regulator of biofilm formation in the presence of low pH [27]. It is important to note that the activation of some TCSs depends on more complex stress-response cascades. Such cascades involve a network of signalling molecules and sensors to start the autophosphorylation that are not directly relevant to host-derived molecules or are reliant on molecules that are produced by the microbiome as well [28]. These TCSs with more complex signalling, though related to pH, are outside the scope of this review and thus not discussed here.

Osmolarity is another gut condition that has many contributing factors. Osmotic stress is linked dysbiotic conditions in the gut, such as inflammatory bowel disease and Crohn’s disease, and osmotic stress can be a marker for the onset of a pathogenic infection, like that seen with *Listeria monocytogenes* infection [93–95]. Osmolarity of the gut is thought to be utilized by some bacterial TCSs as an indication of conditions favourable for infection, triggering the onset of virulent phenotypes [96–98]. The EnvZ-OmpR complex is a well-characterized and widespread TCS known to respond to osmotic conditions. In *E. coli* the TCS is active in medium osmotic conditions and in turn controls the production of the OmpC and OmpF porins, ultimately controlling intracellular access to metabolites [99]. In multiple *Yersinia* species, the OmpR response regulator regulates virulence traits including antibiotic resistance and motility [29]. *Y. pestis*, like many pathogens, has sophisticated iron uptake and regulation mechanisms in order to both evade the host’s immune sequestration of iron and to ensure adequate sources of this central metal ion (see section 4.1 for more on metal ions). OmpR has been identified as one response regulator that directly induces production of the iron-regulating HemR1 protein via binding to the related Phem-1 promoter region [29]. The activation of genes involved in the production of haem receptors as a response to external conditions is invaluable for pathogenic success, as the sense and response mechanisms allows for tighter control and a more attuned virulent response. Though the role of the OmpR regulator in many *Yersenia* species is known, additional work is necessary to confirm the full TCS cascade and to specify the specific environmental cues that activate or repress this cascade.

## 5 Conclusion

The ubiquity of TCSs throughout the genomes of individual members of the gut microbiome community members, combined with the ability of TCS to respond to host cues to regulate virulence, emphasizes the importance of these signalling systems in the context host–microbe interactions. Not only do these systems modulate bacterial stress and metabolism, TCSs also modulate biofilm and virulence traits through diverse mechanisms, making them interesting targets to control bacterial behaviour. Perhaps, the unique control offered by TCSs makes them opportune target for future research aimed at understanding how to modulate bacterial virulence without the use of antibiotics. Metabolic products are known to have roles beyond their direct involvement in basic metabolism, like with tryptophan-derived serotonin, serving as metabolic substrates, host neurotransmitters, and bacterial signalling molecules. The role of metabolic products as signalling molecules for influencing bacterial phenotypes is not novel, but it is an underutilized therapeutic option, especially with the rise in multidrug resistant pathogens. Host-associated molecules like metal ions, amino acids, and amino acid metabolites and host gastrointestinal conditions like ph contribute to the regulation of virulence traits in the gut microbiome via their role as signals for TCSs. Despite their ubiquity, metabolite activated TCSs remain an understudied area, with very few experimentally confirmed whole cascades, which define the entire pathway from signal to sensor to effect. This lack of evidence, in part, stems from the cooperativity and complexity of the initiating ligands that induce overlapping responses. The area of TCS-host metabolite cross talk is a rich one that requires more investigation to clearly define the specific opportunities to use these systems as alternatives to antimicrobial use. Given the multifaceted activities and diverse bioactivity of metabolites produced by both the host and the microbiome, and the established importance of the gut microbiome, it is worth further exploring the expanded role of metabolites and regulators of gastrointestinal conditions as regulators of the microbiome and as contributors to host health.

## Data Availability

All the underlying data were derived from the literature. The analysis for metagenomes were done using publicly available sequences from IMG-M (https://img.jgi.doe.gov/) and described by Nayfach et al. 2020 (DOI:10.1038/s41587-020-0718-6).
